# Beyond ‘Go and bring your husband’: a COM-B guided qualitative study on the barriers to male involvement in antenatal care in Bamenda Health District, Cameroon

**DOI:** 10.1371/journal.pgph.0002904

**Published:** 2025-05-09

**Authors:** Lily Haritu Foglabenchi, Tanya Marchant, Heidi Stöckl

**Affiliations:** 1 Department of Disease Control, London School of Hygiene and Tropical Medicine, London, United Kingdom; 2 Maternal and Child Health Unit, Cameroon Baptist Convention Health Services, Bamenda, Cameroon; 3 Institute for Medical Information Processing, Biometry and Epidemiology, Faculty of Medicine, Ludwig-Maximilians-Universität München, Munich, Germany; University of the Southern Caribbean, TRINIDAD AND TOBAGO

## Abstract

Maternal and infant mortality remains a major public health concern in sub-Saharan Africa. While male involvement in pregnancy and child birth has proven to be an effective intervention that can significantly reduce these deaths, low-income settings like Cameroon continue to encounter obstacles in engaging men in maternal and child health services. This study seeks to examine and contextualize barriers to male partner involvement in antenatal care in order to inform the development of an intervention aimed at promoting male participation in maternal and child health in Cameroon. We employed a qualitative approach drawing upon 68 semi-structured interviews (SSIs) and three focus group discussions (FGDs). This involved purposively selected pregnant women (SSIs-38; FGD-01), male partners (SSIs-30; FGD-01) and health workers (FGD-01). Semi-structured interviews and group discussions were audio-recorded, transcribed and organized using Nvivo. Guided by the Capability, Opportunity and Motivation (COM-B) model of behaviour and Theoretical Domains Framework, we coded and analyzed data using directed content analysis, followed by inductive thematic analysis. Our findings suggest that, the behaviour of expectant fathers during pregnancy is shaped by the dynamic interaction between limited awareness on the role of men in pregnancy care and limited maternal agency to initiate or involve their partners in antenatal care. We also noted that the low motivation of male partners to participate in antenatal care is at the intersection of limiting health system approaches that have not moved beyond mandating women to go and bring their husbands, restrictive gender norms on masculinity—underpinned by the perception that antenatal care is a woman’s affair and the fear of judgement/HIV testing. Based on our findings, we recommend that health system approaches specifically take into account existing knowledge gaps on male partner role, culture-specific gender norms and restructure the health system to promote male participation in maternal and child health services.

## Introduction

Maternal mortality has remained high despite global efforts to promote safe motherhood as laid out in the Sustainable Development Goals (SDG 3.1) [1]. Worldwide, 287,000 women died from pregnancy-related complications in 2020 [2]. Over 99% of these preventable deaths occurred in low-income settings with sub-Saharan Africa (SSA) accounting for over 70% of the global burden of maternal deaths [3]. Evidence suggests that access to and utilization of quality antenatal care and skilled attendance during pregnancy and childbirth can be an effective strategy to improve maternal and child health (MCH) [4]. Male involvement in MCH has therefore been proposed as a crucial strategy in resource-limited settings because male partners have economic and decision-making power and consequently significant influence over the health-seeking behaviours of their pregnant partners [5].

The global recognition of men as key players in MCH has its roots in the 1994 International Conference on Population and Development (ICPD) in Cairo, Egypt [6]. The ICPD program of action laid emphasis on male shared responsibility and participation in sexual and reproductive health as a means of achieving gender equality, equity and women’s empowerment [7]. Research points to the fact that the mechanism through which male involvement impacts maternal and child outcomes is linked to the influence men have over maternal behaviours [8]. Furthermore, data from an intervention study across African countries suggests that the three indexes that consistently determine women’s use of antenatal services and skilled birth attendance (SBA) are: a husband’s involvement in maternal decision-making, spousal discussions, and counselling on birth preparedness [9]. This has recently been supported by a number of studies that suggest that male involvement in maternal health improves the utilization of prenatal and postnatal services, prevents pregnancy complications and improves overall maternal and infant survival [10,11]. Despite these benefits, low levels of male involvement has been reported in SSA with figures as low as 14% in South Africa [12]; 26% in Kenya [13]; 27.1% in Nigeria [14]; and 6-65% in Uganda [15].

Cameroon is currently ranked at the 16^th^ position globally for maternal deaths with an estimated maternal mortality ratio of 438 per 100,000 live births in 2020 [2]. The government developed the 2016-2027 Health Sector Strategy with the aim of advancing and aligning the 2013 multisectoral program with the Sustainable Development Goals targeting 80% of maternal and child health issues both at the community and health facility levels by 2027 [16,17]. Although male involvement is not specifically enshrined in the aforementioned health sector strategy, the National Gender Policy Document (2011-2020) in keeping with the country’s “Vision 2035”, highlights the need for men’s involvement in maternal, reproductive health and HIV/AIDS prevention strategies [18]. This has however not been translated to target male involvement in antenatal service delivery in Cameroon, resulting to only 4.7% of men participating in antenatal care with their pregnant partners [19].

Studies in SSA with Cameroon inclusive have reported barriers to male antenatal attendance, including social or normative beliefs that antenatal care is a female affair, lack of time, negative staff attitude and fear of HIV testing [11,20]. While most of these barriers are reported from the perspectives of women, few studies have reported barriers from the perspectives of men and service providers. Additionally, the theoretical underpinning and contextual influences on male partner behaviour during the antenatal period are not clearly understood. We therefore used the Capability, Opportunity and Motivation (COM-B) Model of Behavioural analysis to contextualise male partner antenatal care behaviour in order to inform the development of an intervention that is aimed at promoting male involvement in maternal and child health in Cameroon.

The COM-B model is central to behaviour change wheel and asserts that a given behaviour (B) can be understood or performed when there is an interaction between Capability (C), Opportunity (O) and Motivation (M) [21]. In this model, capability is defined as the physical (strength, skill or stamina) and psychological capacity (knowledge, skills or innate dispositions) to engage in a behaviour while opportunity could be physical (time, resources) or social—norms and social cues outside an individual that can enable or prompt a behaviour. Motivations are automatic (emotions and impulses) or reflective (evaluations, beliefs, intentions) brain forces that fire and direct behaviour [22]. The COM-B model was suitable for our study because it has an advantage over a single theory, provides a straightforward and comprehensive guide for the categorization and description of factors that impede male partner involvement in antenatal care. Additionally, when mapped to the behaviour change wheel, it offers a systematic pathway for the selection of appropriate behaviour change techniques that could address these determinants [23]. We used COM-B in combination with the Theoretical Domains Framework (TDF) which features 14 domains that further expand on COM-B constructs and captures mediating factors on behaviour change [22,24]. While COM-B has been applied to successfully design interventions in a variety of contexts like medication adherence, smoking cessation, non-communicable diseases and STI testing, limited evidence exists on its use in developing countries or the design of maternal and child health interventions [25,26]. To address the aforementioned gaps, this study applied the COM-B model to explore barriers to male involvement in antenatal care attendance, in order to provide a theoretical understanding of male involvement behaviour and inform intervention development for pregnant couples in the North West Region of Cameroon.

There is currently no agreed definition and indicator for measuring male involvement in MCH. The term varies depending on context—and this ranges from male antenatal care (ANC), immunization or infant welfare attendance, male partner testing for during pregnancy, to spousal discussion and men’s domestic and financial support during pregnancy [27,28]. In this study, we define male involvement as a man attending ANC with his pregnant partner.

## Methods

This study was nested in a larger study that is being conducted to develop an intervention to promote male involvement in MCH/HIV during the prenatal period. It represents the formative phase of the study and data collection took place between January 11th and December 21^st^ 2021. We report this study’s methodology and results following guidance from the Consolidated Criteria for Reporting Qualitative Research (COREQ) [29].

### Study design

We chose a qualitative study design underpinned by the naturalistic enquiry approach to explore perspectives on the barriers to male ANC attendance [30]. This was suitable as it enabled us to use semi-structured interviews (SSIs) and Focus Group Descriptions (FGDs) to capture participant accounts and provide data-rich and comprehensive summaries of the factors that impede male participation in antenatal care.

### Study setting

Our study was conducted in Bamenda Health District in the North West Region of Cameroon.The North-West region is a historically disadvantaged, politically unstable and high-density region—currently ranked as the 3^rd^ most populous in Cameroon with an estimated population of 2 million inhabitants [31]. The region has one of the poorest reproductive, maternal and child health indices in the country. Antenatal care coverage of at least one visit was 58% in 2017; childhood immunization coverage was 68% and under five mortality was 57 per 1000 live births [19,32]. In 2018, the adult HIV prevalence in the region was estimated at 4.0%, which is higher than the national average while maternal HIV prevalence was estimated at 5.0% [33]. The region is largely patriarchal with prohibitive gender norms and socio-cultural customs that impede male involvement in maternal and child health services [34,35].

Bamenda Health District is the largest of the 19 districts in the North West Region. The district is located in an urban and peri-urban locality composed of 17 health areas and 35 health facilities—18 public, 12 lay private and 5 confessional serving an estimated population of 800,000 urban and rural residents [31]. The main public facility is Bamenda Regional Hospital – a level 2 referral hospital. The district covers a total surface area of 560km^2^ and is centrally located within the city of Bamenda —which serves both as the administrative headquarters of Mezam Division and capital city of the North West Region of Cameroon. The city is cosmopolitan with inhabitants originating across the national territory and neighbouring Nigeria. It is made up of three towns: Mankon, Nkwen and Bamendankwe represented by Bamenda I, II, and III Sub-divisions respectively [31].

Study participants were drawn from Nkwen Baptist Hospital within Cameroon Baptist Convention Health Services (CBCHS) —a private faith-based NGO in Cameroon. The hospital has a modern infrastructure with a 114 bed capacity and 250 staff attending to over 18000 patients monthly [36]. It was purposively chosen because it is centrally located within Nkwen town in Bamenda II sub-division, and has high volume antenatal clinics (over 358 clients per month) with well-established Option B+ services. It also attracts a mix of clients with varying socio-cultural backgrounds that was important for this study.

### Participant recruitment and sampling approach

Pregnant or recently postpartum women, male partners and ANC/HIV health workers who were 18 years and above were eligible for inclusion in this study. We employed purposive maximum variation sampling in order to achieve maximal variation regarding age, parity and couple ANC attendance. The variation in our selection was underpinned by the need to capture a wide range of perspectives, perceptions and experiences in order to identify and report common patterns that emerge from heterogeneity [37]. We therefore observed interactions during group antenatal and post-natal consultations to subjectively identify information-rich participants who met our study criteria [38].

We followed recommendations for sample size estimation based on the study question, established evidence on sample size estimates for similar studies and informational redundancy as per the +3 criterion [39,40]. A total of 103 eligible women and their male partners were therefore approached of whom 90 consented; four women declined and nine men who were contacted through telephone were unreachable. Of the 80 pregnant women and male partners who accepted our invitation, 44 were women (n= 38 SSIs; n=6, FGDs) and 36 were men (n= 30 SSIs; n=6, FGDs). We purposefully recruited 10 Staff members (two male and eight female) for an FGD based on their clinical roles within ANC/HIV units and their level of education.

To ascertain that participant perspectives were sufficiently exhausted, we held debriefing sessions to assess, the depth and breadth of participant views, reviewed fieldnotes for recurrent and divergent themes, and noted where infrequent or no new views were expressed on specific research question [41,42].

### Data collection

The data collection process began with site visits for institutional approval, ANC observation, participant recruitment between January and June 2021. This was closely followed by interviews and FGDs interspaced with debriefing sessions between July and December 2021. The corresponding author (LHF)— conducted the majority of SSIs (53) and co-facilitated all FGDs. LHF was assisted by one female Bachelor’s degree nurse midwife and one female master’s level sociologist who served as co-interviewers, observers and note-takers during group discussions. The study’s languages were Pidgin (Cameroonian Creole) and English depending on participant preferences. We took field notes during interviews and FDGs to capture non-verbal cues and unanticipated events. Although these notes served as an additional source of data, they were not used as primary data sources during analysis. They provided context to participant responses, aided debriefing and enriched analytic memos.

SSIs and FGDs were conducted using topic guides (S1 Text). The development of these guides was theoretically underpinned by empirical evidence, the National MCH handbook and the COM-B model mapped unto the TDF. Participants were broadly asked: *What are the barriers that prevent men from attending ANC? Why do you perceive them as barriers? How do you feel about these barriers?* We used these guides iteratively with additional prompts beginning with participants’ responses to enrich and add depth to concepts that emerged during interviews and group discussions.

With the exception of the staff FGD guide, topic guides and demographic forms were piloted with two postpartum women and one pregnant woman. These were further refined following feedback from participants and the research team. With the exception of two SSIs, all SSIs and FGDs were conducted face-to-face in a private room at the health facility. With permission from participants, interviews and FDGs were audio-recorded, translated and transcribed verbatim. Interview sessions lasted between 22 – 65 minutes while FGDs lasted between 1-2hrs. Participants were provided snacks and transport reimbursements which ranged between 350frs CFA (40pence) and £1 (750frs CFA).

### Data management and analysis

Both FGDs and SSIs transcripts were combined to represent one data set and managed using Nvivo Windows (Release 1) © Qualitative Research Solutions International (QSR) [43] and Microsoft programmes ^49^. The first five transcripts were checked against audio recordings for accuracy following the agreed transcription guide that was developed for this study. The overall approach to analysis was directed content analysis followed by inductive thematic analysis [44,45]. Summarily, analysis broadly involved a 7-stage approach with combined guidelines adapted from both Hsieh and Braun as outlined in the codebook (S1 Table)) and Table 1 below [44,45]. The relevance of themes for inclusion was informed by three criteria: (1) frequency of occurrence, (2) presence of conflicting beliefs (3) perceived strength of the belief to influence the target behaviour [46,47].

**Table 1:** Directed Content and Thematic Analysis adapted from Hsieh and Braun [44,45].

Stages	Explanation
**1.Data Familiarisation**	Repeated reading and annotation of transcripts for immersion to gain an intimate knowledge of the data. While reading, key ideas and broad analytic thoughts were generated for later review.
**2.Deductive Coding**	Descriptive labelling of a subset of the data (15 purposively selected transcripts) with succinct tags/codes to identify attributes that were relevant to COM-B, TDF and research questions.
**3.Development of Coding framework**	Categorization or grouping of initial codes to generate contextual descriptions and inferential meanings for later application on the entire dataset. This ended with the development of a COM-B/TDF guided codebook (See S1 Table) that was later applied to the remaining data set for thematic analysis.
**4.Theme Identification**	Inductive application of coding framework on the entire dataset to generate candidate themes and belief statements that represent tentative themes.
**5.Theme Review**	Testing candidate themes against the entire dataset for coherency and consistency in the theme narrative.
**6.Theme Refinement**	Amalgamation and linking of candidate themes into dominant categories. This involved detailed analysis of each theme to decide its name, scope and whether or not it needs to be split or left to stand alone.
**7.Interpretation and Writing up**	Description of themes, reflection and explanation of patterns within themes and reporting with illustrative participant quotes that back up themes.

This was finalized with a tabular representation of themes matched to belief statements, COM-B constructs and relevant theoretical domains. This coding matrix (S2 Table) was reviewed by TN and HS for further insight, refinement, interpretation and exploration of dissonant areas.

### Ethical consideration

Ethical approval for this study was granted by the London School of Hygiene and Tropical Medicine ethics committee (Ref: 18003) and the Cameroon Baptist Convention Health Services Institutional Review Board (IRB2019-33). Written informed consent was sought from all participants. Participants were assured of confidentiality and the non-impact of their participation on the care they were receiving.

### Inclusivity in global research

Additional information regarding the ethical, cultural, and scientific considerations specific to inclusivity in global research is included in the S1 Checklist (Inclusivity-in-global-research-questionnaire)

## Results

Table 2 presents the socio-demographic characteristics of study participants. Over half (56%) were female; 73% were married, 25% were cohabitating while 3% were single. Our respondents had varied occupations, with 36% in professional employment (excluding staff members), 55% reporting self-employment such as bike or taxi driver, farmer, trader and hair dressing. Majority were literate with (37%) having college or university level education. Very few (19%) reported that they had attended ANC with their partners. Among staff members, two were male while eight were female among which we had midwives (06), nutritionist(01), an HIV physician (01), an HIV site nurse(01), an Option B+ team lead(01) a PMTCT regional Focal point nurse(01).

**Table 2:** Socio-demographic characteristics of study participants.

Variable	SSIs (n=68)				Male & Female FGDs (n=12 participants)				Staff FGD (n=10 participants)			
	Female		Male		Female		Male		Female		Male	
	N	%	N	%	N	%	N	%	N	%	N	%
	38	56	30	44.1	6	100	6	100	8	80	2	20
**Marital status**												
Married	29	76	20	67	4	66.7	5	83	--	--	--	--
Cohabiting	7	18	10	33	2	33.3	1	17	--	--	--	--
Single	2	5	--	--	--	--	--	--	--	--	--	--
**Level of Education**												
Primary or less	10	26	7	23	1	16.7	2	33	0		0	
Secondary to high school	13	34	11	37	2	33.3	3	50	3		0	
Completed university	15	40	12	40	3	50	1	17	5		2	
**Employment status** ^*^												
Employed professional	15	40	7	23	3	50	4	67	--	--	--	--
Self-employed	16	42	23	77	3	50	2	33	--	--	--	--
Not working	7	18	---	---	--	--	--	--	--	--	--	--
**ANC Visit with partner** ^**^												
No	30	83	23	77	3	50	4	67	--	--	--	--
Yes	3	8	7	23	3	50	2	33	--	--	--	--
**Mean age**	**31**				**30**				**37**			

* Not working includes students, housewives and not employed; Self-employed includes bike rider, farmers, traders, hairdresser etc;

** Five missing data on ANC attendance with partner; -- not applicable or data not collected.

Five themes were generated from our analysis: (1) limited awareness/knowledge on the need for male involvement, (2) limited female agency to initiate male involvement in ANC, (3) restrictive gender and socio-cultural norms regarding male ANC attendance, (4) limited engagement of male partners by ANC staff and (5) intrapersonal fears that fuel the avoidance of ANC clinics.

We conceptually organised these by mapping them to belief statements and theoretical constructs within the capability, opportunity and motivation domains in the COM-B model as shown in S1 Fig below:

### Limited awareness on male partner role in antenatal care

Several participants reported that the low involvement of men in antenatal care was as a result of limited awareness on their role in maternal and child health and specifically ANC attendance which is generally perceived as a woman’s duty. As noted by participants below, men don’t think they have a part to play during antenatal care and largely perceive antenatal attendance as the duty of their pregnant partners:

*“I think the very first thing is that men fail to know that they have a part to play during antenatal attendance because they think it is the woman’s duty to come, learn and practice what she has been told”—* Male FGD participant, 29yrs.

*“He is not really informed about the importance of couple ANC visit as a parent or partner should” —*Female SSI participant, 31yrs.

A subset of study participants equally echoed this perspective blaming this lack of awareness on the health system —for failing to inform or mandate men on their role or the need to attend antenatal care alongside their partners. For example, a participant chimed during group discussions:

*“I blame the ignorance on the part of the health authorities. They have not made a provision for us. If a pregnant woman does not go for antenatal care, even an uneducated grandmother will ask her why because they know it is mandated. However, when a man does not go, nobody will ask him questions because it is not mandated anywhere. I believe that these health institutions should be the ones to make it mandatory and permit men to have their own clinic day or come along with their wives”.—* Male FGD participant, 34yrs.

### Limited female agency to initiate male involvement

While majority of female participants reported that they don’t have the capability to bear the initial responsibility of convincing and involving their male partners in antenatal activities, providers mandate them to bring their partners for antenatal care. Majority of these participants revealed that they don’t have the agency to bear the initial responsibility of convincing and involving their male partners in antenatal activities. Both male and female respondents therefore expressed the preference for the health system to bear the initial invitation for male involvement. As shown below, this was considered more authoritative than second-hand information passed through pregnant partners.

*“Sometimes I when I tell him that they said men should come for ANC, he thinks I am joking and only want him to be moving around with me. When he sees an invitation from the hospital he would know that it is a serious issue. Otherwise, it will sound like I am the one forcing him to come.”—* Female SSI participant, 27yrs.

In addition to the lack of female agency, respondents opined that some pregnant women were barriers to male participation in antenatal care. Since the financial bulk of pregnancy care is born by men, some women sought to conceal the true cost of antenatal services by preventing their partners from attending ANC in the hope of receiving more money than is actually needed. As outlined by participants below, these women feared that the presence of their partners could expose their extortionary schemes.

*“Women are also barriers. They don’t want their partners to come as participant number 5 mentioned because they don’t want the men to know the amount they are spending for ANC. They use pregnancy as a forum to exhort a lot of money from their partners.”—* Staff FGD participant, 49yrs.

*Some women don’t also give the opportunity for their husbands to come with them for financial reasons. They don’t want them to know what is happening here and how much is being spent. —* Female SSI participant, 36yrs.

### Restrictive gender, social & cultural norms on male ANC attendance

Patriarchal and restrictive gender or cultural norms was a cross-cutting theme that was reported to influence male engagement in antenatal care. Gender normative assumptions that men are superior to women and should opt-out of antenatal care in order to pursue their bread-winning role and dominance were some of the factors that participants echoed for the low participation of men in antenatal care. We constructed sub-themes in this category and substantiated them below with illustrative quotes:

#### Male partner sense of superiority about attending ANC.

During interviews and group discussions, participants reported that the Cameroonian society is largely patriarchal. They noted that men hold positions of dominance and are considered superior to women to the extent that attending antenatal care together with other women was a tacit admission that they are in equal standing with women. Respondents therefore intimated that this perception on the superiority of men deter expectant fathers from attending ANC together with their partners.

*“I think there is a cultural [gender] attachment to this. You know, we African men we have a certain way of relating with women—it is a kind of boss-subordinate relationship. I am the head of the family. I have to dictate and the woman follows…Men don’t see themselves and women as being equals. So they don’t feel comfortable sitting and being given health education together with women. A man may feel like, ‘if I go to clinic with my wife, it may appear like my wife and I are equal’ ” —* Male SSI participant, 39yrs.

#### Lack of male social identity in antenatal clinics.

Participants also reported that men perceive ANC as a woman’s affair because it is largely attended by women who run their own feminine activities which men don’t socially identify with. In the absence of peers or forefathers who modelled antenatal attendance, the few men who attended ANC felt shy, out-numbered and out of place in a large pool of women. As exemplified by male participants below, men question their identity and relevance when they find themselves in a room full of pregnant women during antenatal care:

*The first day I went, I was in a pool of women and all eyes were on me. I was like…OK “what am I doing here?” I am not pregnant! —*Male SSI participant, 35yrs.

*“I am always scared of going for ANC. Imagine being the only man among hundreds of women and the stares you will receive. It makes me feel a type…there are songs that women will be singing, clapping and even dancing to… and they may be expecting you to be clapping and singing as well [laughs]. When you are not clapping because you don’t identify, it becomes a call for concern. When you sing along for solidarity, it is still a call for concern because it may appear as though you are pregnant as well, of which you are not!” —*Male SSI participant, 33yrs.

#### Engendered perception of the value of time and ANC attendance.

The perception that time is not prioritised equally between men and women in Cameroon was reported as an important factor that influence male partner engagement in antenatal care. Participants reported that the majority of Cameroonian men were primary bread-winners—and being able to provide was a source of masculine identity and pride. As echoed by a male participant below, this comes with an expectation for men to appear busy with income-generating activities—not antenatal care which was perceived as an opportunity cost and a liability to men’s bread-winning roles.

*“You have the issue of time…time factor which is also something so important because the man is always busy from morning till night… and remember that it is the man who in most cases (I can say about 80% or 70% of cases), the man is the one who provides everything in the house. So, he makes sure that he catches up with those activities in order to be able to provide. Because when he sits somewhere and loses a day, it is really something big that he has lost” —*Male SSI participant, 33yrs.

A female respondent equally noted that, antenatal attendance is considered an obligatory activity for women but optional for men who don’t wish to forfeit bread-winning for mere antenatal attendance:

*“The point is that coming to the clinic for me is a must. Time or no time I must be at the clinic on the appointed date until delivery. That is not the same for my husband. As a man, he must go out and struggle to work so that he can get something (money) to give me for ANC attendance.” —*Female SSI participant, 31yrs.

#### “Woman Wrapper” stigma—male fear of losing control and female fear of appearing in control.

Since antenatal care is traditionally perceived as an activity reserved for women, the introduction of men into antenatal spaces was largely perceived as an attempt to usurp male dominance. The mental picture projected by respondents on male ANC attendance was a woman at the forefront and the man behind her as a follower. Men who therefore made attempts to accompany their partners for antenatal care were reportedly mocked by community members and ridiculed as weaklings who have given up their power or are under metaphysical forces from women. Additionally, ‘Woman wrapper’*—*which literally translates to being too attached and subdued under a woman’s loin cloth was a prominent term used to mock men who accompany their partners for antenatal care.

*“To add to what my brother has said, let me speak from my community. It appears like, attending ANC with a woman is a way of giving up your authority as a man. When your fellow men see you, they look at you like you are a woman [group laughter]. Yes, you are a woman and not the man because if you were a man, you will not be following your wife to go do women’s things” —*Male FGD participant, 35yrs.

Similarly, the need for men to maintain their dominance over women was observed to be pervasive in the Cameroonian context to the extent that even women who attended antenatal care with their partners reflectively defended their partner’s choice in order to distance themselves from onlooker perceptions which suggest that they are controlling their husbands.

*“You know, African men have a mentality that if they follow their wives for antenatal care, people might see them and think that he lacks something to do or he is a “woman wrapper” [weakling, sissy or subdued man]…The day we came for ANC, he was the only man who came for ANC and even though some women will think that I am controlling my husband, that was his personal decision” —* Female SSI participant, 27yrs.

### Limited engagement of men by ANC staff

Staff and participants who had previously attended antenatal care opined that the engagement of men by ANC staff was ridden with tokenism which some men find derogatory. Others felt their presence in ANC made no difference because the focus was mostly on their pregnant partners. Sub-themes generated from this category are further expanded with the following illustrative quotes from participants:

#### Token-based engagement (ANC claps for men who attend).

Participants reported that men who were spotted in ANC clinics were given a special ‘ANC clap’ to recognize their presence. While this was generally good-intentioned, some men received this with mixed feelings. As noted by a staff member below, some men complained that being clapped for made them uncomfortable and was synonymous to being reduced to preschoolers.

*“Some men have said: “I came to the clinic and at the end they said ‘let’s clap for papa, papa came for clinic today’ and they felt like they were in primary school. So they will not come again, because they don’t like being treated as kids” —*Staff FGD participant 39yrs.

#### Limited male engagement and female-focused health education.

Some participants reported that health facilities have not made provision for their presence in ANC clinics. This is evident in the fact that male attendance has not been required and those who made the effort to attend, providers rarely engaged them. Rather, the entire focus was on their pregnant partners. Some participants further substantiated this with the fact that the need for couple attendance is something they only heard about during our study.

*“You are the first to bring up this procedure on couple ANC attendance and testing for HIV. That is what we should have done before but the attention of the hospital has mostly been on her.” —*Male SSI participant, 42yrs*.*

### Intrapersonal emotions (Fear)

Study participants across FGDs and SSIs reported that male ANC attendance evokes a range of emotions that serve as barriers. These were largely reported as fears and guilt and are substantiated below with participant quotes:

#### Fear of being judged or discovered for extra-marital affairs.

Respondents mentioned that pregnancy in the Cameroonian context opens avenues for men to visit their “deuxième bereau”—local lexicon denoting experimentation with extra-marital relationships. It was therefore opined that some men avoid antenatal clinics because they risked being exposed for their infidelity. Participants equally noted that some aspects of health education during ANC have judgmental undertones that might prick the consciences of men involved in extramarital affairs.

*“Once a woman becomes pregnant, some men get into the practice of what we call ‘side chicks’… they avoid ANC all-together because they don’t want to be judged about their sinful lives and extra-marital affairs. They don’t want health talks at the clinic to echo in their mind and their consciences*” —Female SSI participant, 34yrs.

*“Some men are ashamed especially when they have impregnated many girls in the neighbourhood and they don’t want to be tagged as a particular woman’s husband when there are other women he has impregnated and he is denying being responsibility for their pregnancies.”* —Female SSI participant, 2yrs

#### Fear of HIV testing.

Male involvement in the Cameroonian context is largely driven by the HIV epidemic and need for PMTCT to the extent that participants associated male ANC attendance with HIV testing. As opined by participants below, men feared attending ANC because they will be required to test for HIV.

*“The ANC testing requirement is also a barrier. Perhaps they are aware that if they come, they will be checked and tested and for those who are unaware/unsure of their status, they don’t want to come” —* Male SSI participant, 30yrs.

*“If a woman is coming for ANC and she tells her husband that they are going to do HIV testing for both of them, at that point you will hear the man say ‘my coming is not necessary’. The name HIV alone cancels the whole issue” —*Female SSI participant, 26yrs.

#### Avoidance of responsibility.

In a setting like Cameroon where paternity is rarely established through civil and legal means, male ANC attendance is seen as an explicit form of taking both paternal and financial responsibility. Some respondents reported that men attempt to escape this responsibility by shying away from antenatal attendance. Additionally, the wide practice of extra-marital affairs during pregnancy probably results in pregnancies that most men don’t want to be publicly associated with through ANC attendance.

*“I think there is also a financial barrier. Some men don’t want to attend ANC because attending will mean they are taking responsibility as authors of the pregnancy and this also means they are required to show up as fathers and engage in financial responsibilities”*

*—*Male SSI participant, 39yrs.

## Discussion

This research paper sought to identify and characterize barriers to male partner involvement in antenatal care in the North West Region of Cameroon. Our study findings illustrate the complexity of factors that influence men’s perceptions and behaviour towards ANC attendance. We conceptualised these using the COM-B model of behaviour and TDF with the main barriers reported as: limited awareness on the male role in ANC, female agency to initiate male partner involvement (psychological capability), inadequate engagement of male partners by facility staff, restrictive gender norms on masculinity, the perception that antenatal care is a woman’s affair (social and physical opportunity) and the fear of HIV testing or the related stigma associated with male presence in antenatal clinics (reflective motivation).

The COM-B component of opportunity was highly salient in this study, with social opportunity strongly representing patriarchal perceptions through four thematized barriers: male superiority, lack of male social identity in ANC clinics, engendered perception of time and fear of the ‘woman wrapper’ stigma. Significant among these was the widespread belief that antenatal attendance is a female affair and men who engage in antenatal-seeking behaviours are jobless, “weak” and under the control of their wives. As a result, the participation of men in ANC activities is socially discouraged as it competes with their bread-winning roles and shapes perception on the identity, masculine credibility and engagement of men who venture into antenatal clinics. These gendered perceptions on male ANC attendance has been discussed in a related study [48] and echoed across previous studies in sub-Saharan Africa [49–52]. This current study further generates insight on the multidimensional influence of gender and socio-cultural perceptions on male attendance of antenatal care and underscores the importance of examining its cross-cutting influence over individual motivations, couple dynamics, and institutional approaches that attempt to engage men in pregnancy care. Thus, intervening on this barrier will require the health system to move beyond the instrumentalization of male partners to engaging communities in gender-transformative sensitization and adapting antenatal clinics into spaces that incentivize expectant partners to attend antenatal care.

Physical opportunity took the form of the organizational culture where the health system does not adequately initiate male involvement and fails to engage male partners when they make the effort to attend ANC alongside their pregnant partners. This finding is consistent with other studies in the African sub-continent where the institutional operationalization of male involvement approaches have primarily focused on women—either as information relay agents to their male partners or initiators of their involvement in maternal and child health programs [53,54]. As such, participants in our study proposed that the health system needs to go beyond the traditional *‘go and bring your husband’* agenda by extending a direct appeal to men. While this recommendation is consistent with recent study implications in Malawi and Zambia [55], it is in stark contrast to a study in Tanzania where participants proposed that women should bear the emotional and intellectual burden of involving their partners in maternal and child health [56]. However, this responsibility was observed to be exploited by a minority of women who deterred their partners from attending antenatal care in order to extort additional finances. While this behaviour echoes previous research [57,58], our study presents emerging evidence that centers maternal related barriers on economic reasons—suggesting how the normative expectation for men to be financial providers can facilitate maternal access to pregnancy care while simultaneously fostering their exclusion in clinical care.

Psychological capability was equally a key domain, which underpins male involvement in ANC through limited awareness and knowledge on the need for male partner participation in antenatal activities. Consistent with our study, evidence from studies in South Africa and Ghana demonstrated that the lack of awareness on the need for male involvement influenced male behaviour and non-participation in antenatal care activities [59]. Despite the documentation of high levels of knowledge regarding ANC activities by Nkuoh and colleagues in Cameroon [20], our study demonstrates that this does not necessarily translate to interest or behaviour change on their participation in antenatal care. This disconnect between knowledge and motivation could likely be attributed to the gendered perceptions on the term ‘clinic’ as a female-focused intervention. This equally raises a theoretical and programmatic question: what is the conceptual understanding of male partner awareness and how can the health system bridge the gap between awareness and the involvement of men in pregnancy care?. Addressing this will require a gender-transformative and contextual understanding of awareness-attitude gaps that tailor communication on antenatal education to messaging that engage men directly, reflects need and their inclusivity in the antenatal care package.

Further to inadequate health system engagement and motivation, participants in our study associated the involvement of men in antenatal care with HIV. This might be due to the fact that the structural and conceptual basis of male involvement programs in Cameroon and Sub-Saharan Africa has historically been driven by the HIV epidemic [20,52,60]. Hence, the most common indicator used for measuring male involvement in antenatal care has been prenatal HIV testing ^22,52,60^. Additionally, the fear men expressed for HIV testing and its possible outcome in antenatal settings could be attributed to the prevailing gender norm that men are strong and should therefore not be sick or seen in spaces generally reserved for weak and vulnerable members of the society like women and children. It is against this backdrop that male ANC attendance is highly stigmatised. Thus, the need to go beyond applauding mere ANC attendance or HIV testing to family-based antenatal care and the involvement of men in other clinical assessments like foetal heart monitoring, health education and convenient ANC scheduling. These could potentially reframe the stigmatization of antenatal HIV testing and impact motivation to participate in antenatal activities.

Our study is the first in Cameroon to identify and conceptualise factors that influence male engagement in antenatal care. A unique feature in this study is the use of the COM-B model and TDF as an additional step following thematic analysis to characterise determinants of male involvement in antenatal care. The endorsement of barriers across COM-B components demonstrates the capacity of the model to address limitations of mainstream individual and socio-cognitive theories—by providing a socio-ecological perspective on the complexity of personal, interpersonal and environmental drivers of male partner involvement in antenatal care [61,62]. Additionally, while some behavioural models like the theory of planned behaviour and health belief model only help to understand or predict behaviour, they do not provide systematic pathways for intervention development or behaviour change [63,64]. With COM-B centrally located within the behaviour change wheel, identified factors in our study can be systematically linked to appropriate intervention functions and behaviour change techniques that could address barriers to male involvement in antenatal care [22].

Our study should however be interpreted in light of methodological and practical constraints that limit the generalizability of our findings. First, we noted that specific gender and socio-cultural norms that characterise the North West Region may limit the extent to which our results are generalizable to other regions in Cameroon. Notwithstanding, there was no indication that our findings on the barriers to male involvement in ANC differ significantly from the prevailing literature in Cameroon and Africa at large.

## Conclusion

Our study found that the low motivation of male partners to participate in antenatal care is at the intersection of social and physical opportunities factors (restrictive socio-cultural/gender norms) and psychological capability (limited knowledge and agency). Based on this finding, we recommend that the development of interventions for male involvement in antenatal care engage men directly through gender-transformative and multi-pronged interventions that address structural, socio-cultural and gender norms that restrict their involvement.

## Supporting information

S1 Text Topic guides. (DOCX)

S1 Checklist Inclusivity in global research. (DOCX)

S1 Table Codebook. (DOCX)

S2 Table Coding matrix. (DOCX)

S1 Fig Conceptual analysis of Barriers. (TIF)

## References

[1] UNDESA. Transforming our world: the 2030 Agenda for Sustainable Development | Department of Economic and Social Affairs [Online]. Cited Sep. 06, 2023 Available: https://sdgs.un.org/2030agenda

[2] WHO. Trends in maternal mortality 2000 to 2020 estimates by WHO, UNICEF, UNFPA, World Bank Group and UNDESA/Population Division. Geneva, 2020.

[3] WHO. Maternal mortality, Key Facts [Online]. 2023. Cited Aug. 14, 2023 Available from: https://www.who.int/news-room/fact-sheets/detail/maternal-mortality

[4] Brown CA, Sohani SB, Khan K, Lilford R, Mukhwana W. Antenatal care and perinatal outcomes in Kwale district, Kenya. BMC Pregnancy Childbirth. 2008;8:2. doi: 10.1186/1471-2393-8-2 18186921 PMC2254374

[5] World Health Organisation. WHO recommendations on health promotion interventions for maternal and newborn health. Geneva: WHO; 2015. 26180864

[6] EuroPROFEM. Male Involvement in Reproductive Health: ICPD-Family Care International. Cited Apr. 11, 2023 [Online]. Available: http://www.europrofem.org/contri/2_04_en/en-masc/53en_mas.htm

[7] Lazarus J. The changing role of men since ICPD.Entre Nous Cph Den, Copehagen, Denmark. [Online]. Apr. 1999. Cited Apr. 11, 2023 Available from: https://pubmed.ncbi.nlm.nih.gov/12222325/12222325

[8] Ngom P, Debpuur C, Akweongo P, Adongo P, Binka FN. Gate-Keeping and Women’s Health Seeking Behaviour in Navrongo, Northern Ghana. African Journal of Reproductive Health. 2003;7(1):17. doi: 10.2307/3583341 12816310

[9] Mwakayongwe T. Testing approaches for increasing skilled care during childbirth: Key findings [Online]. 2007. Cited Aug. 14, 2023 Available from: http://www.childinfo.org/areas/deliverycare/countrydata.php

[10] Tokhi M, Comrie-Thomson L, Davis J, Portela A, Chersich M, Luchters S. Involving men to improve maternal and newborn health: A systematic review of the effectiveness of interventions. PLoS One. 2018;13(1):e0191620. doi: 10.1371/journal.pone.0191620 29370258 PMC5784936

[11] Yargawa J, Leonardi-Bee J. Male involvement and maternal health outcomes: systematic review and meta-analysis. J Epidemiol Community Health. 2015;69(6):604–12. doi: 10.1136/jech-2014-204784 25700533 PMC4453485

[12] Yende N, Van Rie A, West NS, Bassett J, Schwartz SR. Acceptability and Preferences among Men and Women for Male Involvement in Antenatal Care. J Pregnancy. 2017;2017:4758017. doi: 10.1155/2017/4758017 28243473 PMC5294384

[13] Aluisio AR, Bosire R, Bourke B, Gatuguta A, Kiarie JN, Nduati R, et al. Male Partner Participation in Antenatal Clinic Services is Associated With Improved HIV-Free Survival Among Infants in Nairobi, Kenya: A Prospective Cohort Study. J Acquir Immune Defic Syndr. 2016;73(2):169–76. doi: 10.1097/QAI.0000000000001038 27124363 PMC5023460

[14] O.-B. A. I., A.-O. E. O., A. A. O., A. A. A., O. S. O. Perception, attitude and involvement of men in maternal health care in a Nigerian community. J Public Health Epidemiol. 2013;5(6):262–70. doi: 10.5897/JPHE2013.0505

[15] Sugiyama Y, Sasajima J, Mizukami Y, Koizumi K, Kawamoto T, Ono Y, et al. Gli2 protein expression level is a feasible marker of ligand-dependent hedgehog activation in pancreatic neoplasms. Pol J Pathol. 2016;67(2):136–44. doi: 10.5114/pjp.2016.61449 27543868

[16] Minsante (Ministry of Public Health). Health Sector Strategy 2016 - 2027 |, Yaounde, Cameroon [Online]. 2017. Cited Apr. 17, 2023 Available from: https://www.minsante.cm/site/?q=en/content/health-sector-strategy-2016-2027-0

[17] Minsante (MOH). National Multisector Program to Combat Maternal, Newborn and Child Mortality (PLMI-CMR) [Online]. Ministry of Health, Cameroon, 2013. Cited Apr. 21, 2023 Available from: https://www.plmi.cm/

[18] Japan International Cooperation Agency (JICA). 2015 Country Report of Gender Profile (Cameroon). 2015.

[19] NACC. PMTCT Progress Report [Online]. 2017. Available from: http://www.cnls.cm/sites/default/files/rapport_progres_ptme_ndeg_12_2017_1.pdf

[20] Nkuoh GN, Meyer DJ, Tih PM, Nkfusai J. Barriers to men’s participation in antenatal and prevention of mother-to-child HIV transmission care in Cameroon, Africa. J Midwifery Womens Health. 2010;55(4):363–9. doi: 10.1016/j.jmwh.2010.02.009 20630363

[21] Michie S, van Stralen MM, West R. The behaviour change wheel: a new method for characterising and designing behaviour change interventions. Implement Sci. 2011;6:42. doi: 10.1186/1748-5908-6-42 21513547 PMC3096582

[22] Michie S, Atkins L, West R. The Behaviour Change Wheel: A Guide to Designing Interventions. 2014.

[23] Atkins L, Francis J, Islam R, O’Connor D, Patey A, Ivers N, et al. A guide to using the Theoretical Domains Framework of behaviour change to investigate implementation problems. Implement Sci. 2017;12(1):77. doi: 10.1186/s13012-017-0605-9 28637486 PMC5480145

[24] Cane J, O’Connor D, Michie S. Validation of the theoretical domains framework for use in behaviour change and implementation research. Implement Sci. 2012;7:37. doi: 10.1186/1748-5908-7-37 22530986 PMC3483008

[25] Han S, Middleton PF, Bubner TK, Crowther CA. Women’s views on their diagnosis and management for borderline gestational diabetes mellitus. J Diabetes Res. 2015;2015:209215. doi: 10.1155/2015/209215 25785278 PMC4345277

[26] Fulton EA, Brown KE, Kwah KL, Wild S. StopApp: Using the Behaviour Change Wheel to Develop an App to Increase Uptake and Attendance at NHS Stop Smoking Services. Healthcare (Basel). 2016;4(2):31. doi: 10.3390/healthcare4020031 27417619 PMC4934584

[27] Mkandawire E, Hendriks SL. A qualitative analysis of men’s involvement in maternal and child health as a policy intervention in rural Central Malawi. BMC Pregnancy Childbirth. 2018;18(1):1–12. doi: 10.1186/S12884-018-1669-5 29351778 PMC5775573

[28] Ditekemena J, Koole O, Engmann C, Matendo R, Tshefu A, Ryder R, et al. Determinants of male involvement in maternal and child health services in sub-Saharan Africa: a review. Reprod Health. 2012;9:32. doi: 10.1186/1742-4755-9-32 23171709 PMC3573948

[29] Tong A, Sainsbury P, Craig J. Consolidated criteria for reporting qualitative research (COREQ): a 32-item checklist for interviews and focus groups. Int J Qual Health Care. 2007;19(6):349–57. doi: 10.1093/intqhc/mzm042 17872937

[30] Sandelowski M. Whatever happened to qualitative description? Res Nurs Health. 2000;23(4):334–40. doi: 10.1002/1098-240x(200008)23:4<334::aid-nur9>3.0.co;2-g 10940958

[31] Communication Service at the Governor’s Office, North-West Region’s Web Portal [Online]. Cited Feb. 25, 2022 Available from: http://www.northwest-cameroon.com/home-35-front-0.html

[32] The DHS Program - Cameroon: DHS, 2018 - Cameroon 2018 Demographic and Health Survey - Summary Report (English) [Online]. 2018. Cited Apr. 21, 2023 Available from: https://dhsprogram.com/publications/publication-sr266-summary-reports-key-findings.cfm

[33] Minsante (Ministry of Public Health). Cameroon Population-based HIV Impact Assessment (CAMPHIA) 2017-2018: Final Report [Online]. Yaounde, Dec. 2020. Cited Feb. 25, 2022 Available: https://phia.icap.columbia.edu/wp-content/uploads/2021/09/53059-CAMPHIA-Report_EN_WEB_August1.pdf

[34] Nkeneh YC. The Role of Women in the Political Transition of the Bamenda Grassfields Fonship Institutions in Cameroon. Int J Humanit Soc Sci Educ. 2023;10(9):20–9. doi: 10.20431/2349-0381.1009003

[35] Monteh R. Women and Traditional Politics in the Bamenda Grassfields-Cameroon from the Precolonial To Postcolonial Periods. IAR Journal of Humanities and Cultural Studies [Online]. Cited Jan. 20 2020, 2024:39–47. Available from: https://www.iarconsortium.org/article/download/175/

[36] Cameroon Baptist Convention Health Services. Nkwen Baptist Health Center-Overview [Online]. Cited Feb. 25, 2022 Available from: https://cbchealthservices.org/health-centers/north-west-region/nkwen-baptist-hc/

[37] Palinkas LA, Horwitz SM, Green CA, Wisdom JP, Duan N, Hoagwood K. Purposeful Sampling for Qualitative Data Collection and Analysis in Mixed Method Implementation Research. Adm Policy Ment Health. 2015;42(5):533–44. doi: 10.1007/s10488-013-0528-y 24193818 PMC4012002

[38] Creswell JW, Plano Clark VL. Designing and conducting mixed methods research. International Student Edition; 2018.

[39] Marshall B, Cardon P, Poddar A, Fontenot R. Does sample size matterin qualitative research? A review of qualitative interviews in is research. 2013.

[40] Francis JJ, et al. What is an adequate sample size? Operationalising data saturation for theory-based interview studies. 2010. 10.1080/08870440903194015 20204937

[41] Saunders B, Sim J, Kingstone T, Baker S, Waterfield J, Bartlam B, et al. Saturation in qualitative research: exploring its conceptualization and operationalization. Qual Quant. 2018;52(4):1893–907. doi: 10.1007/s11135-017-0574-8 29937585 PMC5993836

[42] Varpio L, Ajjawi R, Monrouxe LV, O’Brien BC, Rees CE. Shedding the cobra effect: problematising thematic emergence, triangulation, saturation and member checking. Med Educ. 2017;51(1):40–50. doi: 10.1111/medu.13124 27981658

[43] QSR International, “NVivo Windows (Release 1),” 2020, 1.7.1 [Online]. [Cited May 31, 2024]. Available from: https://help-nv.qsrinternational.com/20/win/Content/welcome.htm#

[44] Hsieh H-F, Shannon SE. Three approaches to qualitative content analysis. Qual Health Res. 2005;15(9):1277–88. doi: 10.1177/1049732305276687 16204405

[45] Braun V, Clarke V. Using thematic analysis in psychology. Qualitative Research in Psychology. 2006;3(2):77–101. doi: 10.1191/1478088706qp063oa

[46] Francis JJ, Stockton C, Eccles MP, Johnston M, Cuthbertson BH, Grimshaw JM, et al. Evidence-based selection of theories for designing behaviour change interventions: using methods based on theoretical construct domains to understand clinicians’ blood transfusion behaviour. Br J Health Psychol. 2009;14(Pt 4):625–46. doi: 10.1348/135910708X397025 19159506

[47] Gale NK, Heath G, Cameron E, Rashid S, Redwood S. Using the framework method for the analysis of qualitative data in multi-disciplinary health research. BMC Med Res Methodol. 2013;13:117. doi: 10.1186/1471-2288-13-117 24047204 PMC3848812

[48] Foglabenchi LH, Stöckl H, Marchant T. ‘I am a father but not pregnant’—a qualitative analysis of the perspectives of pregnant couples on male partner role during pregnancy care in Bamenda, Cameroon. 2024. doi: 10.21203/rs.3.rs-4155069/v1 PMC11665113 39716286

[49] Kwambai TK, Dellicour S, Desai M, Ameh CA, Person B, Achieng F, et al. Perspectives of men on antenatal and delivery care service utilisation in rural western Kenya: a qualitative study. BMC Pregnancy Childbirth. 2013;13:134. doi: 10.1186/1471-2393-13-134 23800139 PMC3691751

[50] Greene ME, Barker G. Masculinity and Its Public Health Implications for Sexual and Reproductive Health and HIV Prevention.

[51] Craymah JP, Oppong RK, Tuoyire DA. Male Involvement in Maternal Health Care at Anomabo, Central Region, Ghana. Int J Reprod Med. 2017;2017:2929013. doi: 10.1155/2017/2929013 29362725 PMC5736899

[52] Morfaw F, Mbuagbaw L, Thabane L, Rodrigues C, Wunderlich A-P, Nana P, et al. Male involvement in prevention programs of mother to child transmission of HIV: a systematic review to identify barriers and facilitators. Syst Rev. 2013;2:5. doi: 10.1186/2046-4053-2-5 23320454 PMC3599633

[53] Takah NF, Kennedy ITR, Johnman C. The impact of approaches in improving male partner involvement in the prevention of mother-to-child transmission of HIV on the uptake of maternal antiretroviral therapy among HIV-seropositive pregnant women in sub-Saharan Africa: a systematic review and meta-analysis. BMJ Open. 2017;7(11):e018207. doi: 10.1136/bmjopen-2017-018207 29175889 PMC5719335

[54] Nkuoh GN, Meyer DJ. An assessment of strategies used to encourage male involvement in antenatal care and women’s uptake of PMTCT services in Cameroon. African Journal of Midwifery and Women’s Health. 2016;10(1):34–41. doi: 10.12968/ajmw.2016.10.1.34

[55] Mweemba O, Zimba C, Chi BH, Chibwe KF, Dunda W, Freeborn K, et al. Contextualising men’s role and participation in PMTCT programmes in Malawi and Zambia: A hegemonic masculinity perspective. Glob Public Health. 2022;17(9):2081–94. doi: 10.1080/17441692.2021.1964559 34375155

[56] Maluka S, Japhet P, Fitzgerald S, Begum K, Alexander M, Kamuzora P. Leaving no one behind: using action research to promote male involvement in maternal and child health in Iringa region, Tanzania. BMJ Open. 2020;10(11):e038823. doi: 10.1136/bmjopen-2020-038823 33191255 PMC7668372

[57] Ganle JK, Dery I, Manu AA, Obeng B. “If I go with him, I can’t talk with other women”: Understanding women’s resistance to, and acceptance of, men’s involvement in maternal and child healthcare in northern Ghana. Soc Sci Med. 2016;166:195–204. doi: 10.1016/j.socscimed.2016.08.030 27569661

[58] Roudsari RL, Sharifi F, Goudarzi F. Barriers to the participation of men in reproductive health care: a systematic review and meta-synthesis. BMC Public Health. 2023;23(1):1–37. doi: 10.1186/S12889-023-15692-X 37143008 PMC10158256

[59] Ganle JK, Dery I. “What men don’t know can hurt women’s health”: a qualitative study of the barriers to and opportunities for men’s involvement in maternal healthcare in Ghana. Reproductive Health. 2015;12(1):1–13. doi: 10.1186/S12978-015-0083-Y 26452546 PMC4600282

[60] Manjate Cuco RM, Munguambe K, Bique Osman N, Degomme O, Temmerman M, Sidat MM. Male partners’ involvement in prevention of mother-to-child HIV transmission in sub-Saharan Africa: A systematic review. SAHARA J. 2015;12:87–105. doi: 10.1080/17290376.2015.1123643 26726756

[61] Noar SM. An interventionist’s guide to AIDS behavioral theories. AIDS Care. 2007;19(3):392–402. doi: 10.1080/09540120600708469 17453575

[62] Briscoe C, Aboud F. Behaviour change communication targeting four health behaviours in developing countries: a review of change techniques. Soc Sci Med. 2012;75(4):612–21. doi: 10.1016/j.socscimed.2012.03.016 22541798

[63] Ajzen I. The theory of planned behavior. Organizational Behavior and Human Decision Processes. 1991;50(2):179–211. doi: 10.1016/0749-5978(91)90020-t

[64] Becker MH. The health belief model and personal health behavior. Slack; 1974.

