# Supplementary material for: Beyond ‘Go and bring your husband’: a COM-B guided qualitative study on the barriers to male involvement in antenatal care in Bamenda Health District, Cameroon
Source: PLOS Glob Public Health. 2025 May 9;5(5):e0002904. doi: 10.1371/journal.pgph.0002904 (PMC12063873; doi:10.1371/journal.pgph.0002904)
Supplement: S1 Text — (DOCX) [file pgph.0002904.s001.doc]

**TOPIC GUIDES for Semi-Structured Interviews and Focus Group Discussions**

**Development of a Maternal and Child Health/HIV Text-messaging Intervention to Improve Couple Antenatal attendance and HIV Testing in Cameroon**

**OUTLINE**

1. Introduction about the study (purpose, procedure, anticipated benefits, reporting of findings, informed consent, permission to record etc)
2. Rapport Building: Mainly demographic information
3. Interview proper
4. Closing of interview (thanking the participant; reminding them about commitments for confidentiality and privacy from research team)
5. **Perceptions and behavior norms on male partner involvement in antenatal care and HIV-prevention during pregnancy**

**Semi-Structured Interviews**

**Barriers to male ANC attendance**

- Has your partner accompanied you for ANC OR have you accompanied your partner for antenatal care? Why? Why not?
- Do you discuss what happened in ANC with your partner? Why? Why not?
- What are the barriers that prevent men from attending ANC? Why do you perceive them as barriers? How do you feel about these barriers?.

**Focus Group Discussions**

- Do men in your community accompany their partners for antenatal care? If not, why?
- What are the barriers that prevent men from attending ANC? Why do you perceive them as barriers? How do you feel about these barriers?.
