# Supplementary figures and images for: Beyond ‘Go and bring your husband’: a COM-B guided qualitative study on the barriers to male involvement in antenatal care in Bamenda Health District, Cameroon
Source: PLOS Glob Public Health. 2025 May 9;5(5):e0002904. doi: 10.1371/journal.pgph.0002904 (PMC12063873; doi:10.1371/journal.pgph.0002904)

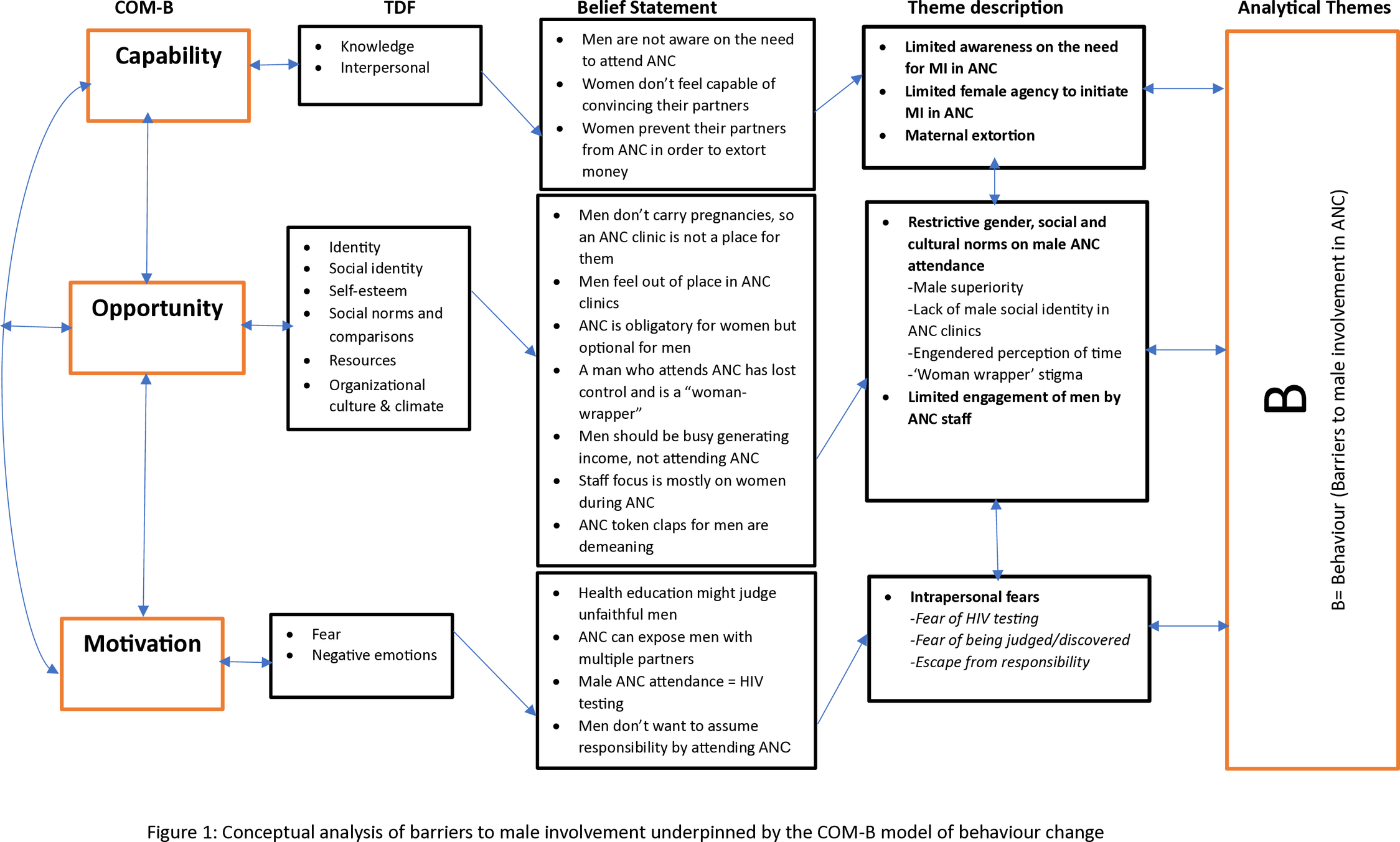

Supplement: S1 Fig — (TIF) [file pgph.0002904.s005.tif]
